# Serologic Evidence for MERS-CoV Infection in Dromedary Camels, Punjab, Pakistan, 2012–2015

**DOI:** 10.3201/eid2303.161285

**Published:** 2017-03

**Authors:** Muhammad Saqib, Andrea Sieberg, Muhammad Hammad Hussain, Muhammad Khalid Mansoor, Ali Zohaib, Erik Lattwein, Marcel Alexander Müller, Christian Drosten, Victor Max Corman

**Affiliations:** Department of Clinical Medicine and Surgery, University of Agriculture, Faisalabad, Pakistan (M. Saqib, M.H. Hussain);; University of Bonn Medical Centre, Bonn, Germany (A. Sieberg, M.A. Müller, C. Drosten, V.M. Corman);; Animal Health Research Center, Ministry of Agriculture and Fisheries, Muscat, Oman (M.H. Hussain, M.K. Mansoor);; Key Laboratory of Special Pathogens and Biosafety, Wuhan Institute of Virology, Chinese Academy of Sciences, Wuhan, China (A. Zohaib);; EUROIMMUN AG, Lübeck, Germany (E. Lattwein);; German Centre for Infection Research, Bonn (C. Drosten, V.M. Corman)

**Keywords:** Middle East respiratory syndrome, MERS-CoV, viruses, coronavirus, animal reservoir, zoonoses, antimicrobial resistance, neutralizing antibodies, serology, One Health, livestock, RNA viruses, dromedary, Bactrian, camels, Pakistan, Asia, Africa

## Abstract

Dromedary camels from Africa and Arabia are an established source for zoonotic Middle East respiratory syndrome coronavirus (MERS-CoV) infection among humans. In Pakistan, we found specific neutralizing antibodies in samples from 39.5% of 565 dromedaries, documenting significant expansion of the enzootic range of MERS-CoV to Asia.

The Middle East respiratory syndrome coronavirus (MERS-CoV) is a zoonotic pathogen that causes severe respiratory disease in humans. Dromedary camels (*Camelus dromedarius*), which have 1 hump on their backs, have been identified as an animal reservoir and source of human MERS-CoV infection (*1*). Reports document widespread infection of these camels on the Arabian Peninsula and parts of Africa (*2*–*4*). Besides these regions, dromedaries are also native to several countries in Asia. A study in Kazakhstan found no evidence for MERS-CoV infection in dromedaries (*5*). Absence of MERS-CoV infection in dromedary camels in Asia could mean a vulnerable animal reservoir at risk for de novo introduction by sporadic contact (e.g., by trade) with dromedaries from MERS-CoV endemic areas. Pakistan is 1 of 8 countries globally, and the only country outside Africa, that has a dromedary population exceeding 1 million animals (FAOSTAT database; http://faostat3.fao.org). Because of the limited capacities for routine MERS-CoV surveillance and a considerable human population size in countries in northeastern Asia, targeted investigation of the MERS-CoV infection status is of interest for global public health agencies.

In this study, we examined dromedaries from Pakistan for exposure to MERS-CoV. We tested 565 serum samples, which we collected from 348 female and 217 male animals by using a convenience sampling strategy in 9 districts of Punjab, eastern Pakistan, during 2012–2015. The median age of the animals was 5 years.

The testing algorithm comprised 2 antibody detection methods (*3*,*6*). MERS-CoV IgG was detected by using a MERS-CoV camel antibody ELISA (EUROIMMUN, Lübeck, Germany). We tested all serum samples that exceeded a cutoff of 0.4, validated in previous studies (*3*,*6*), by using a microneutralization (MN) test for confirmation (*4*). Only serum samples with neutralizing activity >1:80 were considered MERS-CoV antibody–positive.

A total of 315 (55.8%) of 565 camel serum samples exceeded the ELISA signal cutoff (Table). Of these, 223 (39.5%) were confirmed by using MN. We identified MERS-CoV neutralizing antibodies in camels sampled in all study years and in nearly all regions except the district of Chiniot (Table). The rate of neutralizing antibody–positive camel samples ranged from 82.9% in Rahim Yar Khan to 24.1% in the Jhang district (Table).

**Table:** Detection of Middle East respiratory syndrome coronavirus antibodies in dromedary camels from different districts of Punjab Province, Pakistan, 2012–2015*

District	Year of sampling†	No. animals tested	ELISA			MN	
No. positive	Detection rate, % (95% CI)	No. positive	Detection rate, % (95% CI)
Chiniot	2012, 2014	23	4	17.4 (5–38.8)		0	0 (0.0–14.8)
Faisalabad	2012, 2013, 2015	66	45	68.2 (55.6–79.1)		32	48.5 (36.0–61.1)
Jhang	2012, 2013, 2015	220	79	35.9 (29.6–42.6)		53	24.1 (18.6–30.3)
Bhakkar	2012	88	51	57.9 (47–68.4)		36	40.9 (30.5–51.9)
Layyah	2012	13	9	69.2 (38.6–90.9)		4	30.8 (9.1–61.4)
Muzaffargarh	2012, 2013	40	26	65 (48.3–79.4)		15	37.5 (22.7–54.2)
Bahawalpur	2015	29	26	89.7 (72.6–97.8)		21	72.4 (52.8–87.3)
Lodhran	2014	51	42	82.4 (69.1–91.6)		33	64.7 (50.1–77.6)
Rahim Yar Khan	2012	35	33	94.3 (80.8–99.3)		29	82.9 (66.4–93.4)
Total		565	315	55.8 (51.5–59.9)		223	39.5 (35.4–43.6)

*Serum samples were tested at a dilution of 1:100; MNT was done in a microtiter plate format in duplicate at a dilution of 1:80. ELISA ratio >0.4 and MN >1:80 were considered positive. MN, microneutralization. †Samples from all years, except the samples from 2012 and 2014 from Chiniot, were MN positive.

By using a merged dataset comprising all regions, we correlated seropositivity to animal age and sex. Sex-dependent differences suggested pronounced seropositivity in male animals, but differences were not significant (p>0.067; Technical Appendix Table). Seropositivity increased with age: samples from more than half (51.1%) of all animals >5 years of age and less than one third (29.2%) of animals <2 years of age tested positive (p<0.001; online Technical Appendix Table). These age-dependent differences are similar to those found in previous studies (*6*,*7*) and can be explained by long-lasting immune response or regular re-exposure after initial MERS-CoV infection. The finding of antibodies in young camels born in Pakistan suggests ongoing circulation of MERS-CoV in the country.

Bactrian camels (*C. bactrianus*), which have 2 humps on their backs, are also native to Central and East Asia. Studies of these camels in China and Mongolia, as well as dromedary and Bactrian camels in southern Kazakhstan, uniformly reported absence of MERS-CoV during 2014–2015 (*5*,*8*,*9*). However, dromedaries may become sources of infection for Bactrian camels that are susceptible to MERS-CoV and present in this vast geographic range. The possibility of cross-species transmission within the order of camelids has been documented by a study that found signs of MERS-CoV infection in alpacas (*Vicugna pacos*) that shared a barn with dromedaries in Qatar (*10*). The absence of MERS-CoV in camelid populations in northeastern parts of Asia (Mongolia, Kazakhstan, and China) is compatible with the view that the spread of MERS-CoV from Africa into Asia may be a recent development. However, other explanations, including resistance to infection by Bactrian camels, are possible. Studies of susceptibility should be conducted to clarify whether Bactrian camels in Asia could act as a naïve reservoir population in the future.

Based on MERS-CoV antibodies in dromedary camels, our data suggest a risk for human exposure in Punjab, Pakistan, that is similar to risks in Africa and the Arabian Peninsula. Of note, Punjab shares a border with the state of Rajasthan in India, which harbors that country’s largest dromedary population. A similar risk for human exposure is likely for this part of India. However, findings of antibodies against MERS-CoV in migrant workers from these areas should be interpreted with caution because these workers often employed in Arabian countries. For Pakistan, our data largely exclude the scenario of a widely susceptible animal reservoir population in which de novo introduction of MERS-CoV could start an epizootic that could lead to spillover epidemics among humans.

Technical Appendix Univariate analysis of potential risk factors associated with Middle East respiratory syndrome coronavirus seropositivity in dromedary camels in Punjab, Pakistan.
