# Supplementary material for: Serologic Evidence for MERS-CoV Infection in Dromedary Camels, Punjab, Pakistan, 2012–2015
Source: Emerg Infect Dis. 2017 Mar;23(3):550–1. doi: 10.3201/eid2303.161285 (PMC5382745; doi:10.3201/eid2303.161285)
Supplement: Technical Appendix — Univariate analysis of potential risk factors associated with Middle East respiratory syndrome coronavirus seropositivity in dromedary camels in Punjab, Pakistan. [file 16-1285-Techapp-s1.pdf]

Article DOI: http://dx.doi.org/10.3201/eid2303.161285

Serologic Evidence for MERS-CoV Infection in Dromedary Camels, Punjab, Pakistan, 2012–2015

Technical Appendix

Technical Appendix Table. Univariate analysis of potential risk factors associated with MERS-CoV seropositivity in dromedary camels of Punjab, Pakistan

Variable	Category	No. tested	No. positive	Prevalence, %	95% CI	Odds ratio (95% CI)	p value
Sex	Male	217	96	44.2	37.5–51.1	1.38 (0.98–1.95)	$\chi^2 = 3.36$ ($p = 0.067$)
	Female	348	127	36.5	31.4–41.8	1	
Age	>10 Years	88	43	48.9	38.1–59.8	2.23 (1.25–4.30)	$\chi^2 = 25.51$ ($p < 0.001$)
	5.1–10 Years	180	92	51.1	43.6–58.6	2.53 (1.47–4.36)	
	2.1–5 Years	208	62	29.8	23.7–36.5	1.03 (0.60–1.77)	
	≤2 Years	89	26	29.2	20.1–39.8	1	
